# The Quality and Characteristics of Digital Mental Health Apps: Mixed Methods Study

**DOI:** 10.2196/67944

**Published:** 2026-05-11

**Authors:** Maciej Marek Zych, Raymond Bond, Maurice Mulvenna, Jorge Martinez Carracedo, Lu Bai, Tim Andrews

**Affiliations:** 1 School of Computing, Ulster University, 2-24 York Street, Belfast, BT15 1AP, United Kingdom, 44 07526852505; 2 School of Electronics, Electrical Engineering and Computer Science, Queen's University Belfast, Belfast, United Kingdom; 3 ORCHA, Daresbury, United Kingdom

**Keywords:** digital mental health, mental health apps, machine learning, health informatics, data privacy, mobile health

## Abstract

**Background:**

There are around 20,000 mental health apps available in app stores. The Organisation for the Review of Care and Health Apps (ORCHA), a United Kingdom digital health compliance company, has assessed a number of digital mental health apps with regard to their quality, professional and clinical assurance, data privacy, and user experience. This study analyzes the data that were collected by ORCHA when they assessed mental health apps.

**Objective:**

This study aimed to examine the characteristics of mental health apps regarding their quality, target users, features, underpinning evidence, and data privacy.

**Methods:**

A dataset comprising ORCHA Baseline Review assessments of over 2000 digital health apps, including 436 mental health apps, was used. This study uses exploratory data analysis to gain insight into the quality and characteristics of mental health apps. Methods such as descriptive and inferential statistics, k-modes clustering, and association rule mining were used to explore the quality of mental health apps as well as reveal insights into the different cost types, target users, app features, data types, and evidence of app content.

**Results:**

Information provision, data capture, and data sharing were the most common features within the 436 mental health apps. The examined apps primarily targeted the following groups: adults (n=229, 52.5%), everyone (n=184, 42.2%), and teens (n=135, 31%). The cost of apps has not been linked to the quality of mental health apps, although paid apps or apps with in-app purchases may include additional services. Indicated user acceptance or benefit is the most common type of evidence provided by these mental health apps. A total of 241 (55.3%) apps included a qualified professional in app development, and 251 (57.6%) apps provided evidence within the app that the developer validated any guidance with relevant reliable information sources or references. Usage data and email were the most commonly collected data types. Association rule mining showed that email, IP address, name, and usage data are often co-collected by the same apps. K-modes cluster analysis showed that mental health apps can be categorized into 2 clusters, where one cluster of apps (n=182, 41.7%) collected more data than apps in the other cluster.

**Conclusions:**

Mental health apps are commonly targeted for everyone to use, but many apps are targeted toward teens or adults. Our study suggests that many publicly available mental health apps did not take the precautions (such as the involvement of appropriate health professionals, literature references, or conducting tests) to ensure that their content is valid and research based. Greater effort on behalf of mental health app developers is needed to ensure that the public is provided with high-quality apps. Moreover, our study indicates that the mental health apps that collect more data tend to score better on the ORCHA Baseline Review assessment.

## Introduction

According to a World Health Organization report [1], there are nearly 1 billion people living with a diagnosed mental health condition, and most do not have access to effective care. Moreover, during the COVID-19 pandemic, mental health issues became more predominant and prevalent [2-4] and have remained prevalent as of 2024 [1]. Mental health conditions affect many people’s lives; for example, a study from 2020 [5] showed that social stigma is a contributing factor to unemployment in people living with mental health issues. With the decline in mental health, it is important to mitigate a global mental health crisis.

While apps might be useful, it is important to understand their quality. For example, a study from 2020 has shown that college counseling centers in the United States often recommend unsafe and not up-to-date mental health apps to students [6]. Another paper from 2016 [7] has shown that suicide prevention apps may also provide potentially harmful content. Moreover, Sander et al [8] indicated that the public can have difficulty identifying high-quality apps for posttraumatic stress disorder. Beyond apps, other digital tools also require further investigation; for example, social media has been linked to an increase in mental health issues [9,10].

Arguably, the most accessible tools for mental health care in the public are mental health apps or websites [11]. Estimates show that there are around 20,000 mental health apps available in app stores [11,12]. However, it is important to identify mental health apps based on their quality before health care professionals recommend them to patients or clients.

The Organisation for the Review of Care and Health Apps (ORCHA) [13] is a United Kingdom–based digital health compliance company that specializes in the quality assessment of digital health apps. ORCHA is currently working with 70% of National Health Service organizations within England and provides digital health app libraries, hosted by various health care organizations, that contain information about digital health apps and their quality. The ORCHA Baseline Review (OBR) is a proxy for digital health apps’ compliance with best practice standards. Specifically, the OBR assessment results provide information (including an assessment score between 0 and 100) regarding a digital health app’s compliance with best practices in the domains of professional and clinical assurance (PCA), data privacy (DP), and user experience (UX), allowing end users and clinical professionals to make informed decisions on whether to use or recommend these digital health apps. National Institute for Health and Care Excellence (NICE) [14] provides guidance on digital health app research. ORCHA uses Evidence Standard Framework (ESF) [15], developed by NICE, to categorize digital health apps, and the ESF has been influential in the development of the OBR assessment tool.

It has been suggested that mental health apps can enhance but not replace existing mental health services [16]. Similarly to a 2023 study [16], a meta-analysis from 2021 [17] studied the treatment of depression using apps and found that there was no difference in the outcomes between human-guided digital interventions and face-to-face psychotherapy. For apps to do more good than harm, it is important to recommend only mental health apps of sufficient quality for public use. A meta-review of meta-analyses from 2020 stated that more studies related to the quality of mental health apps are needed [18]. Previous studies have assessed the UX, engagement, and popularity of mental health apps [19,20]. This paper also extends the work conducted in 2023 [21], which examined the quality of digital health apps across different health care domains or categories.

Expanding on these studies, the objective of this study was to examine the characteristics of mental health apps, specifically regarding their quality, the level of underpinning evidence, and aspects related to DP. ORCHA has provided a dataset of OBR results and documentation from their assessment of 436 digital mental health apps. While there are studies regarding the use of conversational agents for mental health [22,23], these were not included in this study. To achieve our objective, the following research questions were examined:

1. How do mental health apps score on the OBR assessment tool (as indicated by ORCHA, PCA, UX, and DP scores)?
2. Is there a difference in ORCHA, PCA, UX, or DP scores among mental health apps that are free and those that are paid or contain in-app purchases?
3. Who are the target users of mental health apps?
4. What features are included in mental health apps, and which are linked to the ORCHA score?
5. What evidence is presented to validate the content of mental health apps?
6. What are mental health apps’ use and development characteristics?
7. What data are collected by mental health apps?

By answering the abovementioned questions, we believe we will be able to broadly report on the characteristics of digital mental health apps. This will allow us to identify areas of concern where improvements can and should be made regarding digital health app quality.

## Methods

### The Dataset and Tools

ORCHA provided an assessment dataset of 2127 digital health apps. All the apps were assessed using the OBR tool, Version 6 (OBR V6). The assessment and app categorization of all the digital health apps were conducted by 2 ORCHA reviewers. A third reviewer was included only if there was any dispute between the original 2 reviewers during an assessment. It takes around 6 months for an ORCHA reviewer to be trained on how to use the OBR tool and to be considered ready to carry out live reviews using the tool. The training involves teaching the new reviewer about each area (UX, PCA, and DP) of the OBR. Training is carried out either in person or via online meetings. The dataset used included digital health app assessments that were published between January 18, 2021, and January 6, 2022.

From the 2127 digital health apps, a subset of 436 mental health apps was created and used for this study. The only selection criterion was that an app had been categorized as a “mental health app.” Multimedia Appendix 1 states more specific categories (mutually inclusive) of mental health apps that were included.

The OBR V6 tool is the latest version of the ORCHA assessment tool, which consists of approximately 300 objective (mostly dichotomous yes/no) assessment questions in 3 domain areas: PCA, DP, and UX. Each of the areas is scored individually on a scale from 0 to 100 and combined into an overall ORCHA score. Moreover, the collected data of OBR assessments of mental health apps included information such as target users, features, and associated cost types (free, paid, and in-app purchases). Information relating to app development was also collected, such as whether there is a suitably qualified professional involved in the development team of the app. All the collected data allowed us to examine what sort of evidence is being provided regarding the content of mental health apps.

NICE, a UK-based organization, provides guidance relating to health technologies, including health apps. NICE has produced an ESF that can be used to classify digital health apps using A, B, and C tiers based on their functionality, risk, and regulatory status. All the assessed mental health apps were assigned a NICE ESF tier. Tier A indicates that the digital health apps provide health and social care services with no measurable user outcome. Tier B denotes that the digital health apps can provide a 2-way communication between users and health care professionals and provide health care information or a health diary. Tier C indicates that digital health apps provide preventative behavioral change aimed at health issues; they may allow users to self-manage a specific condition, indicate that the digital health apps provide or guide treatment for a condition, record and transmit data about this condition to a professional, caregiver, or third party without a user’s input, contain a calculator that impacts treatment, provide diagnostics for a specific condition, or guide a diagnosis. Since the NICE tiers are dependent upon functionality, risk, and regulatory status, it would be inappropriate to, for example, use OBR on a tier C digital health app using OBR tier B score weighting. See Checklist 1 for included topics and items in this paper.

### Data Analysis

Descriptive statistics were used to study the data. In this study, box plots, line plots, and heatmaps were used for the exploratory visualization of data. Descriptive statistics were used when conducting analysis of OBR scores, cost types, target users, features, presented evidence, use and development characteristics, and collected data types. Quantiles were used to represent the spread of domain (OBR) scores from 0% to 100% in increments of 25%. Median and IQR were calculated for some of the data.

The Wilcoxon rank sum test was used to compare 2 samples of data to determine any statistically significant differences between them. A *P* value less than or equal to .05 was considered statistically significant. A Bonferroni-corrected α value was calculated when multiple hypothesis testing was conducted. This was used when comparing ORCHA scores between apps that had a specific feature and those that did not.

A random forest algorithm was used to determine feature importance, with mean square error (increase in mean squared error) and node purity (increase in node purity) calculations, when predicting the overall ORCHA score when using app features as predictors (eg, goal setting, gamification).

Association rule mining (a priori algorithm) was used to identify commonly co-occurring types of data collected by mental health apps. K-modes cluster analysis was performed on the data types. K-modes was chosen as a clustering method because the data were recorded in binary format. An optimal number of clusters was determined using the NbClust package in R [24]. The package uses 30 indices to determine the best number of clusters for a dataset.

### Ethical Considerations

This secondary data analysis study gained ethical approval from Ulster University (Ethics Filter Committee, Faculty of Computing, Engineering and the Built Environment, project number: CEBE_RE-22‐002). The process undertaken by ORCHA ensures that digital health app developers are aware of their score and are given time to contest findings of the assessment, which may be amended if developers provide additional relevant information. All reviews, unless explicitly requested to be removed by the developer, are covered as suitable for research in ORCHA’s privacy policy [25]. Informed consent was obtained by ORCHA to use the data from the assessments for the purposes of research, provided that the assessed apps are anonymized in publications.

## Results

### App Selection

**Figure 1.**
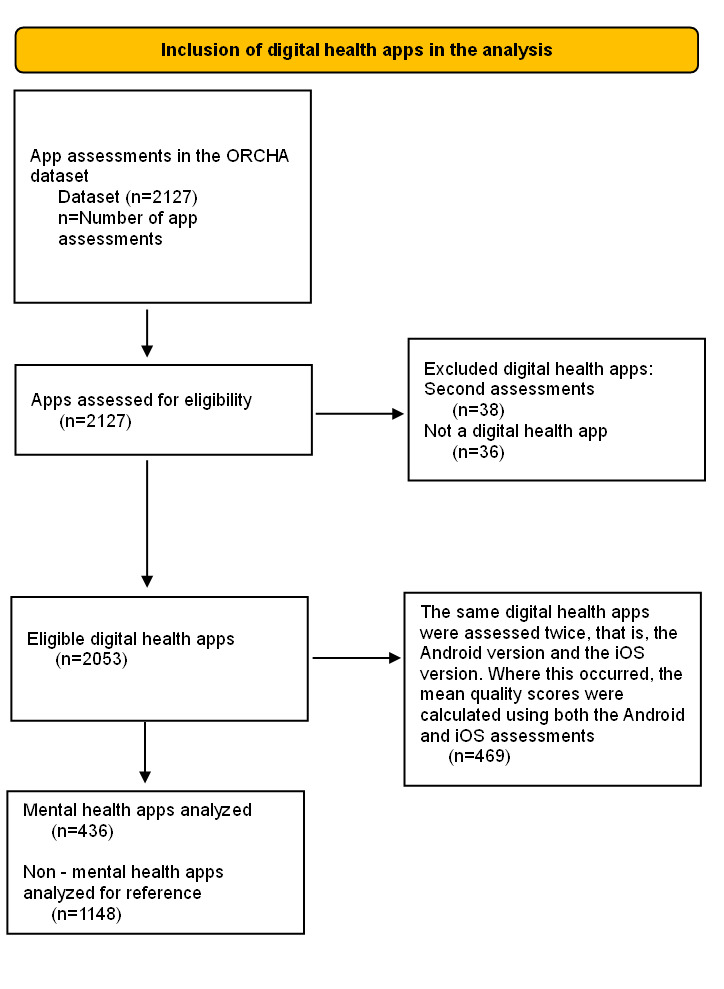
Mental health app selection diagram. ORCHA: Organisation for the Review of Care and Health Apps

Figure 1 shows the process of selecting mental health apps from the ORCHA dataset.

### How Do Mental Health Apps Score on the OBR Assessment Tool (as Indicated by ORCHA, PCA, UX, and DP Scores)?

Table 1 shows domain scores per NICE ESF tier. Color coding was applied as follows: red for values greater than 75, green for values from 65 to 75, and blue for values less than 65. Since there were only 2 mental health apps assigned to tier A, they were not included in Table 1.

As can be seen in Table 1, the median ORCHA score for 436 mental health apps was 60 (Tier B: 62 and Tier C: 55). ORCHA, together with the National Health Service, determined that an ORCHA threshold score of 65 indicates adequate compliance with the assessment criteria to be recommended for use by the public. Most mental health apps do not meet that threshold. This finding demonstrates that greater effort is needed on behalf of developers to produce higher-quality mental health apps.

**Figure 2.**
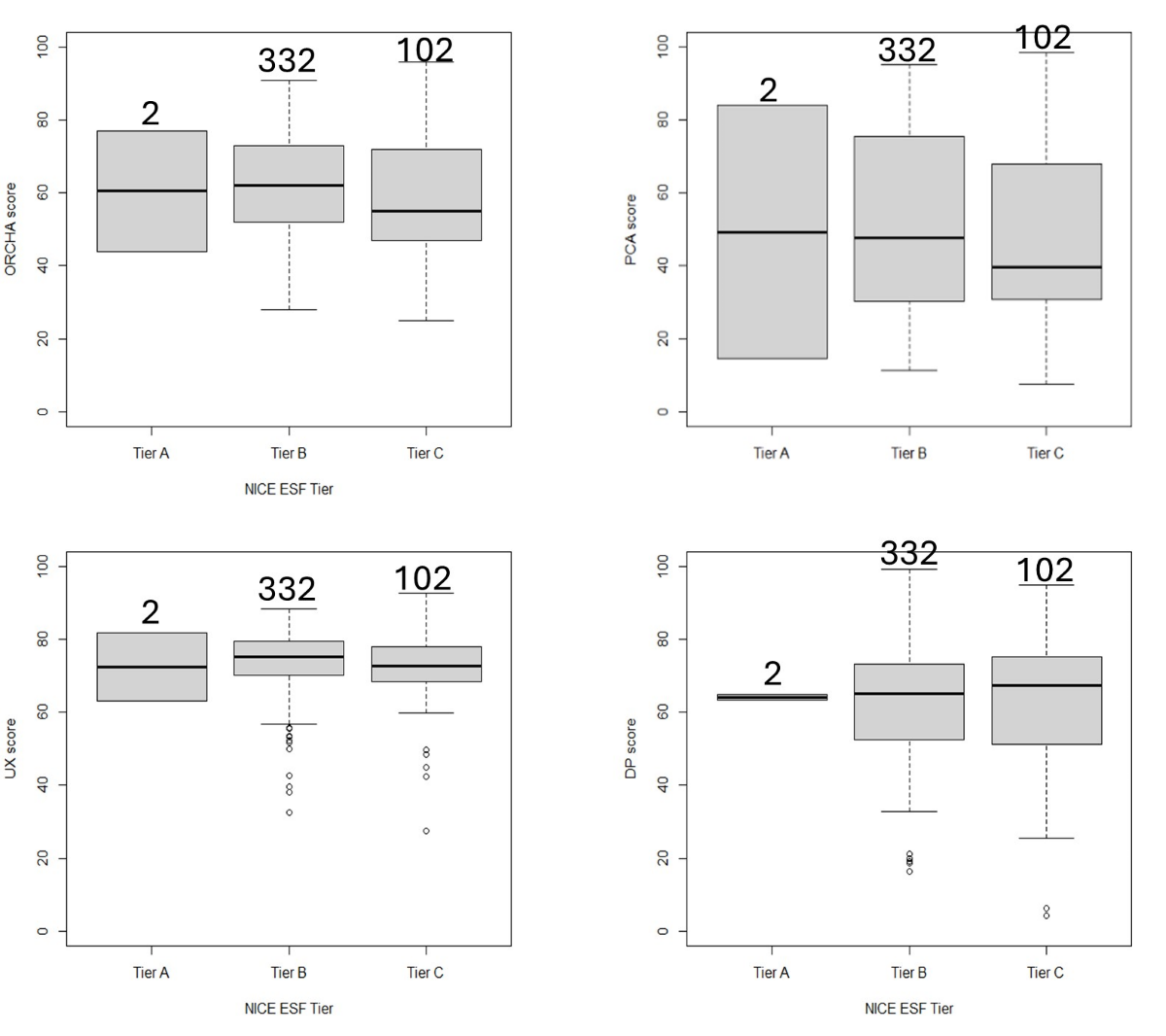
Quality scores per assessment domain (user experience [UX], data privacy, and professional clinical assurance) and per National Institute for Health and Care Excellence (NICE) Evidence Standard Framework (ESF) Tier (Tier A, B, and C). DP: data privacy; ORCHA: Organisation for the Review of Care and Health Apps; PCA: professional and clinical assurance.

Figure 2 visualizes NICE ESF tiers per domain score with box plots. When separated into NICE ESF tiers, the sample sizes were as follows: tier A, 2; tier B, 332; and tier C, 102.

**Table 1.** Mental health apps’ ORCHA scores per tier.

Tier and scores	Values, median (IQR; range)
All apps (n=436)	
ORCHA^a^	60 (51-73; 25.0-96.0)
UX^b^	75.2 (69.8-79.6; 27.4-92.7)
PCA^c^	46.1 (30.3-75.3; 7.57-98.5)
DP^d^	65.6 (52.5-73.1; 4.28-99.3)
Tier B (n=332)	
ORCHA	62 (52-73; 28.0-90.5)
UX	75.2 (70.2-79.6; 32.6-88.3)
PCA	47.7 (30.3-75.3; 11.4-95.3)
DP	65 (52.5-73.1; 16.5-95.3)
Tier C (n=102)	
ORCHA	55 (46.3-71; 25.0-96.0)
UX	72.5 (68.6-77.8; 27.4-92.7)
PCA	39.5 (31.1-68; 7.57-98.5)
DP	67.3 (51.7-75.3; 4.28-94.9)

a ORCHA: Organisation for the Review of Care and Health Apps.

b UX: user experience.

c PCA: professional and clinical assurance.

d DP: data privacy.

### Is There a Difference in ORCHA Score, PCA, UX, or DP Among Mental Health Apps That Are Free and Those That Are Paid or Contain In-App Purchases?

**Figure 3.**
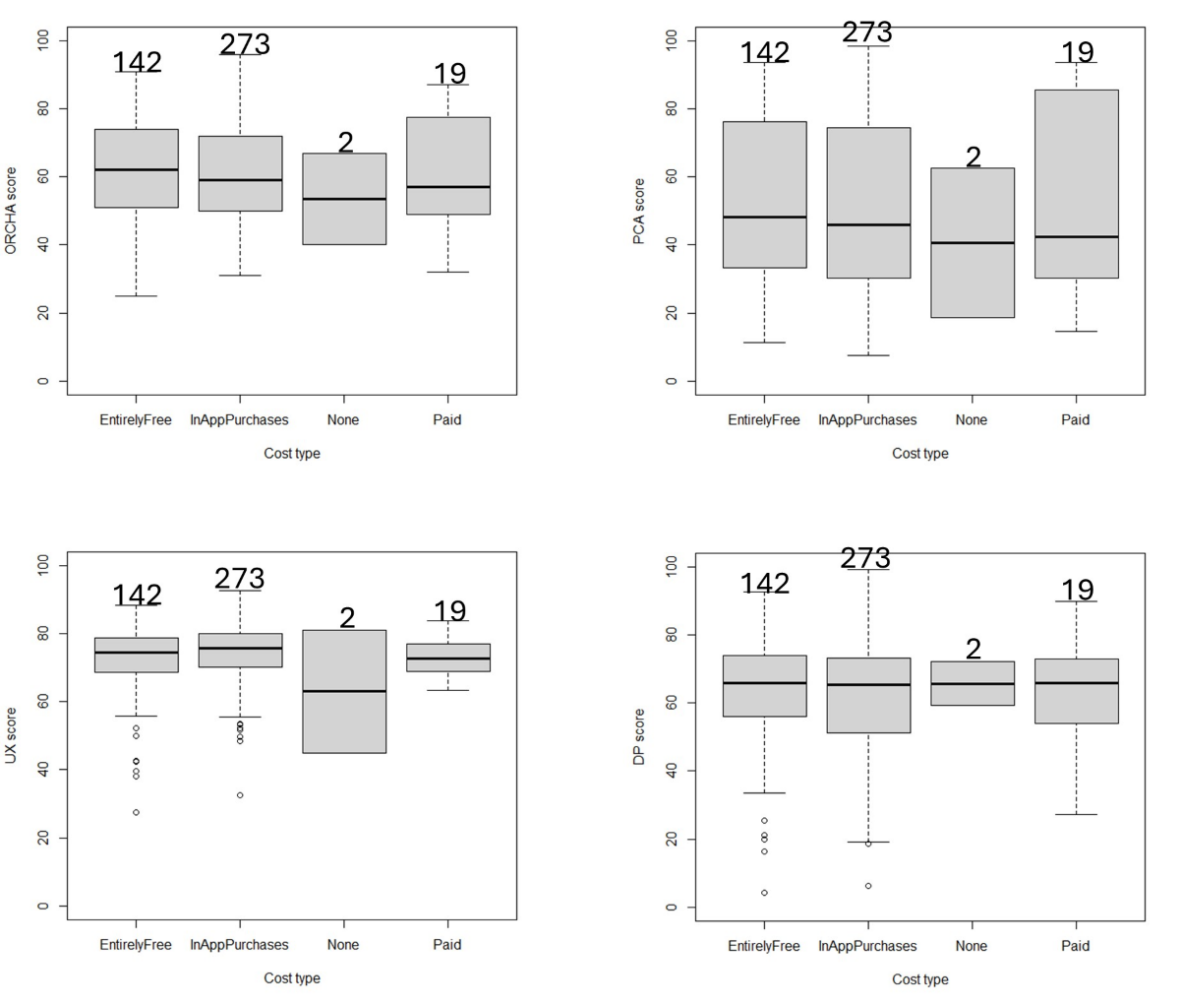
Cost analysis box plots per domain score. DP: data privacy; ORCHA: Organisation for the Review of Care and Health Apps; PCA: professional and clinical assurance; UX: user experience.

Figure 3 shows domain scores against cost type. As can be seen from the box plots, the quality of a mental health app does not appear to be influenced by cost. This is further confirmed by the Wilcoxon rank sum test, where none of the cost types achieved a statistically significant (*P*≤.05) difference when compared for each of the domain scores. When separated by cost types, the sample sizes were as follows: none: 2, paid: 19, entirely free: 142, and in-app purchases: 273.

This study has shown that there appears to be no difference in OBR scores in relation to cost. Figure 3 shows that the distributions of OBR scores (ORCHA, PCA, UX, and DP) for mental health apps that are free, paid, and those with in-app purchases are similar, with a nonstatistically significant (*P*≤.05) difference when using the Wilcoxon 2-sample rank sum test. Nevertheless, it is possible that paid apps or those with in-app purchases may include features not offered by those apps that are available for free. Further study is needed to determine whether paid apps or those with in-app purchases offer features or services that are unavailable in free digital mental health apps.

### Who Are the Target Users of Mental Health Apps?

Table 2 shows the target users of the 436 mental health apps that were included in this study. Target users are presented in descending order of frequency. The table shows that most mental health apps appear to be targeted at adults, followed by everyone and teens.

**Table 2.** Mental health apps’ target users (N=436).

Target users	Frequency, n (%)
Adults	229 (52.5)
Everyone	184 (42.2)
Teenagers	135 (31)
Older adults	51 (11.7)
Children	27 (6.19)
Preteens	20 (4.59)
Clinicians	5 (1.15)
Patients	2 (0.459)
Infants	2 (0.459)
Health care administrators	1 (0.229)
Caregivers	1 (0.229)

### What Features Are Included in Mental Health Apps and Which Are Linked to ORCHA Score?

In this section, we aim to identify features that may improve the use and quality of digital mental health apps. Table 3 shows the features that were recorded for the 436 mental health apps included in this study and their frequency. Features are presented in descending order of frequency. The table indicates that the most common features are information provision, followed by data capture and data sharing.

**Figure 4.**
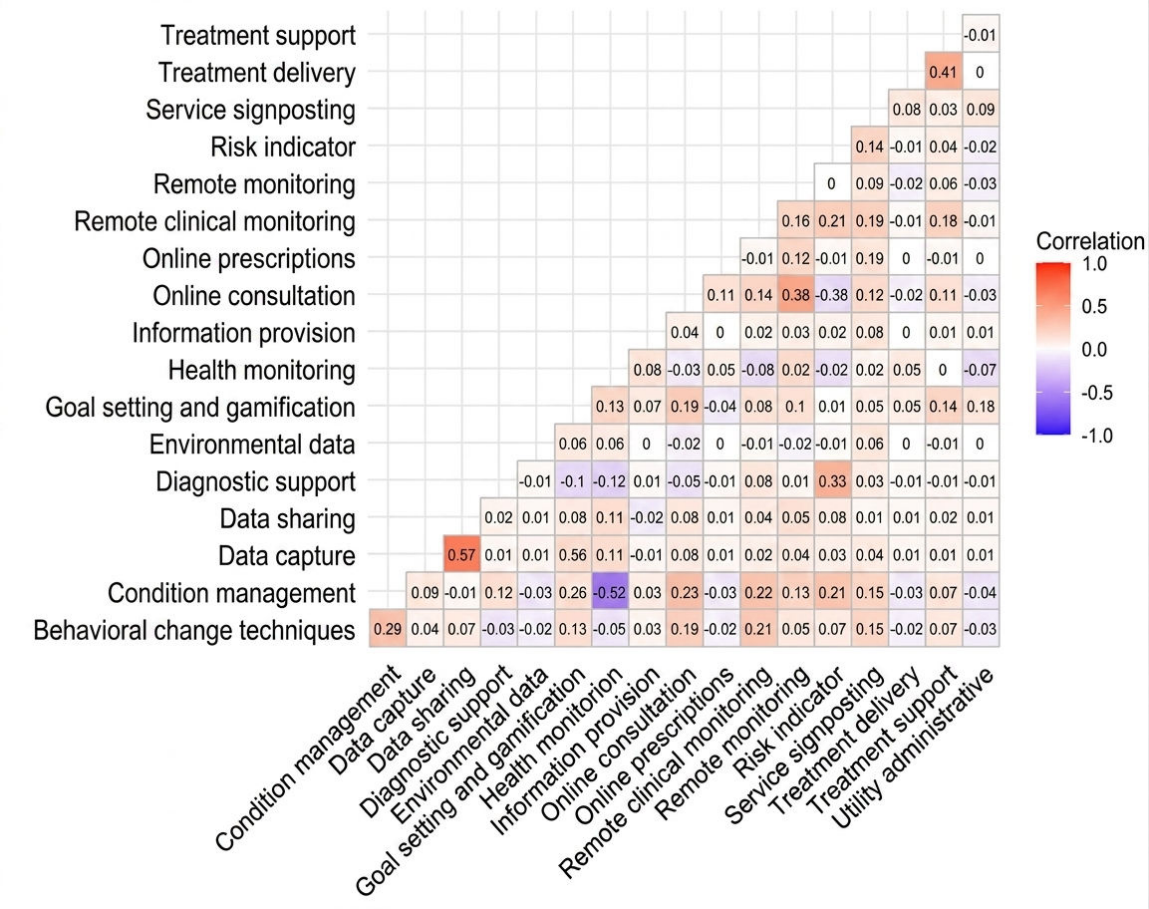
Spearman correlations among features.

**Figure 5.**
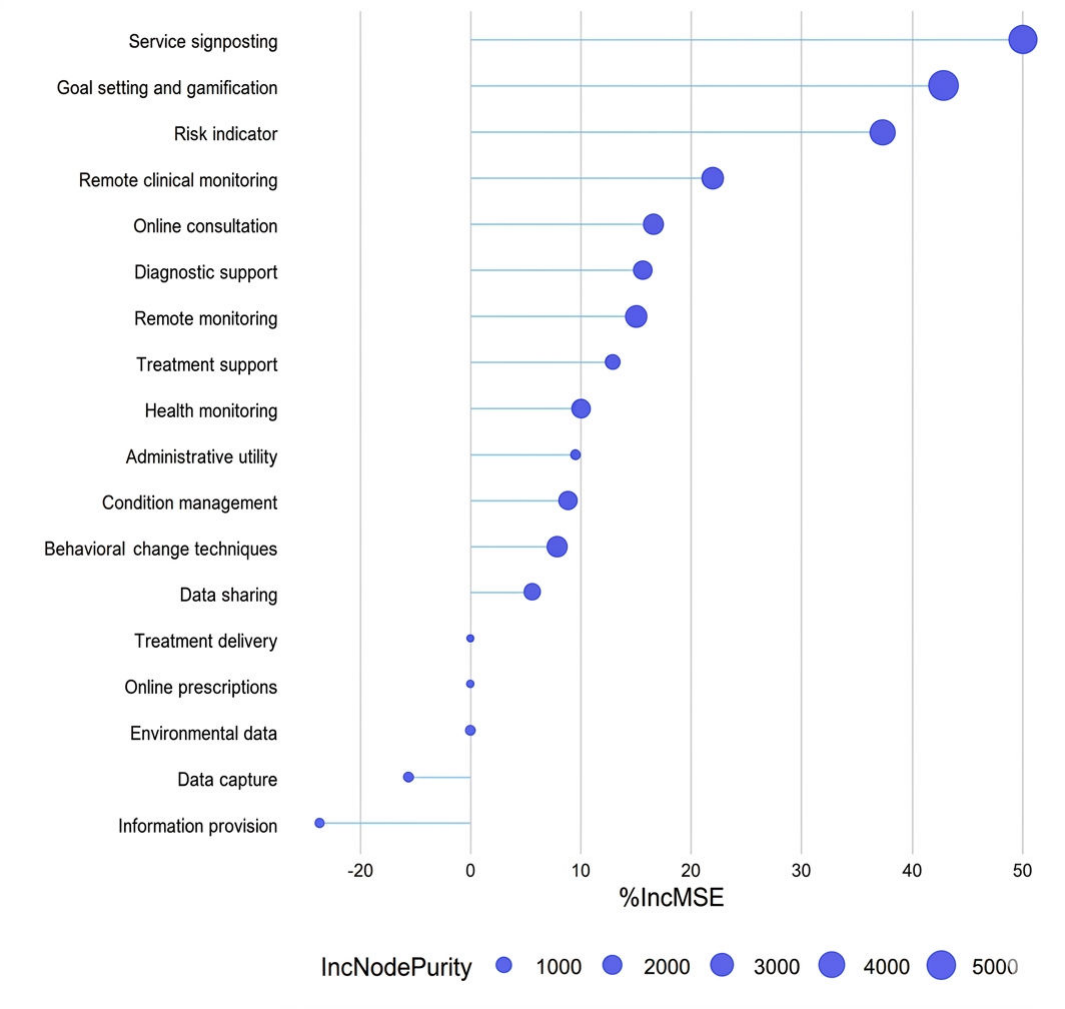
Feature importance with random forest for the Organisation for the Review of Care and Health Apps (ORCHA) score. incMSE: increase in mean squared error; incNodePurity: increase in node purity.

Figure 4 depicts pairwise Spearman correlations among the features. The highest being between data capture and data sharing, with a Spearman correlation of 0.57. Figure 5 depicts the importance of features (increase in mean squared error and increase in node purity), with ORCHA scores as the independent variable and features as the dependent variables. Multimedia Appendix 2 depicts feature importance and correlation for each tier.

**Table 3.** Feature frequency (N=436).

Features	Frequency, n (%)
Information provision	433 (99.3)
Data capture	431 (98.9)
Data sharing	421 (96.6)
Health monitoring	215 (49.3)
Goal setting and gamification	184 (42.2)
Service signposting	159 (36.5)
Condition management	107 (24.5)
Online consultation	69 (15.8)
Remote monitoring	62 (14.2)
Behavioral change techniques	56 (12.8)
Risk indicator	34 (7.80)
Remote clinical monitoring	17 (3.90)
Treatment support	6 (1.38)
Diagnostic support	6 (1.38)
Utility/administrative	2 (0.459)
Treatment delivery	1 (0.229)
Online prescriptions	1 (0.229)
Environmental data	1 (0.229)

Random forest feature importance was used to identify which features are linked to the ORCHA score (Figure 5). Service signposting and goal setting and gamification were identified as the 2 most important features that are positively associated with the ORCHA score, whereas the risk indicator was negatively associated with the ORCHA score.

Table 4 depicts the top 3 features for mental health apps altogether and when partitioned by NICE ESF tier (tier B and tier C). It also states the number of apps with and without the feature, with their ORCHA scores’ median and IQR. The Wilcoxon rank sum test with a Bonferroni-corrected α value was calculated to check for statistically significant differences between the 2 distributions of apps regarding the ORCHA score among apps with and without the feature. See MultimediaAppendices 2  3 for more information regarding features for each tier and data visualizations.

**Table 4.** Top 3 features for each tier. Bonferroni corrected α value is .05/9 ≈ .006.

Tier and feature	Apps with feature, n	Apps with feature ORCHA^a^ score, median (IQR)	Apps without feature, n	Apps without feature ORCHA score, median (IQR)	Wilcoxon test (*P* value)	Effect size (Cohen W)
All apps						
Service signposting	159	66 (55.0-76.5)	277	56 (48.0-71.0)	<.001	0.271
Goal setting and gamification	184	67.5 (54.0-78.0)	252	57 (48.0-68.0)	<.001	0.156
Risk indicator	34	49.5 (45.3 -57.0)	402	61 (51.0-74.0)	<.001	0.844
Tier B						
Service signposting	105	70 (59.0-77.0)	227	57 (49.0-70.0)	<.001	0.368
Goal setting and gamification	127	68 (54.5-78.0)	205	60 (49.0-70.0)	<.001	0.235
Online consultation	38	67 (60.5-74.0)	294	61 (51.0-73.0)	.01	0.771
Tier C						
Goal setting and gamification	57	62 (52.0-76.0)	45	51 (46.0-57.0)	<.001	0.118
Risk indicator	33	48 (45.0-57.0)	69	57 (51.0-76.0)	<.001	0.353
Behavioral change techniques	32	72 (55.0-82.0)	70	53 (46.0-59.5)	<.001	0.373

a ORCHA: Organisation for the Review of Care and Health Apps.

### What Evidence Is Presented to Validate the Content of Mental Health Apps?

Table 5 depicts the types of evidence that were presented in the OBR assessment of mental health apps, separated by NICE ESF tier, and by their medical device status in Table 6. Some mental health apps can be classified as medical devices if they conduct diagnoses or provide treatments. The data indicate that indicated user acceptance (295/436, 67.7%) and observational studies (59/436, 13.5%) are the most commonly presented evidence types for the content of digital mental health apps. However, 118 of 436 (27.1%) digital mental health apps provided no evidence.

Tables 5 and 6 show that the most common evidence presented by mental health apps during the assessment is indicative of user acceptance or benefit (295/436, 67.7%). The rarest type of evidence presented was meta-analysis or systematic review (10/436, 2.29%). More evidence from academic and clinical sources may improve the quality of mental health apps. Relying mainly on user acceptance or benefit does not verify that the content of those apps is of clinical standard and value. Moreover, 118 out of 436 (27.1%) apps did not present any evidence. In the time of a mental health crisis and the accessibility of mental health apps, it should be a requirement for all mental health apps to include a qualified professional in the app development lifecycle, as well as validate any guidance provided by an app with relevant information sources and references to mitigate any risk associated with its use.

**Table 5.** Evidence presented in descending order of frequency per National Institute for Health and Care Excellence Evidence Standard Framework tier.

Tier and evidence type	Frequency, n (%)
Tier A (n=2)	
None	1 (50)
Survey	1 (50)
Observational	1 (50)
Tier B (n=332)	
Indicated user acceptance/benefit	229 (69)
None	96 (28.9)
Observational	35 (10.5)
Pilot study	23 (6.93)
Randomized controlled trial	18 (5.42)
Survey	13 (3.92)
Meta-analysis/systematic review	4 (1.20)
Tier C (n=102)	
Indicated user acceptance/benefit	66 (64.7)
Observational	23 (22.5)
None	21 (20.6)
Pilot study	16 (15.7)
Randomized controlled trial	14 (13.7)
Meta-analysis/systematic review	6 (5.88)
Survey	4 (3.92)

**Table 6.** Evidence presented in descending order of frequency per medical device.

Device and evidence type	Frequency, n (%)
Not a medical device (n=414)	
Indicated user acceptance/benefit	282 (68.1)
None	110 (26.6)
Observational	56 (13.5)
Pilot study	38 (9.18)
Randomized controlled trial	27 (6.52)
Survey	16 (3.86)
Meta-analysis/systematic review	10 (2.42)
Medical device (n=22)	
Indicated user acceptance/benefit	13 (59.1)
None	8 (36.4)
Randomized controlled trial	5 (22.7)
Observational	3 (13.6)
Survey	2 (9.09)
Pilot study	1 (4.55)
Meta-analysis/systematic review	0 (0)

Table 7 depicts the characteristics of evidence that were provided. The table presents which methods were used to validate mental health apps’ evidence, separated by NICE ESF tiers. The key takeaway from the table appears to be that most mental health apps did not involve a qualified professional, and most of the presented evidence did not involve any calculation of the *P* value, as it was deemed not applicable (NA). Table 7 shows that many of the mental health apps in this study did not provide information on the characteristics of the evidence, such as details on the sample size used, use of a comparator, or *P* values, indicating that even when evidence is presented for mental health apps, important related information is missing.

**Table 7.** Evidence characteristics across tiers.

Characteristics	Tier A (apps: n=2), n (%)	Tier B (apps: n=332), n (%)	Tier C (apps: n=102), n (%)
Is there a suitably qualified professional involved in the development team of the app?	Yes: 1 (50); No: 1 (50)	Yes: 169 (50.9); No: 163 (49.1)	Yes: 71 (69.6); No: 31 (30.4)
Is the sample size appropriate?	Yes: 1 (50); No: 0 (0%); N/A^a^: 1 (50)	Yes: 35 (10.5); No: 11 (3.31); N/A: 286 (86.1)	Yes: 23 (22.5); No: 7 (6.86); N/A: 72 (70.6)
Does the evidence found provide a *P* value?	Yes: 1 (50); No: 0 (0); N/A: 1 (50)	Yes: 36 (10.8); No: 10 (3.01); N/A: 286 (86.1)	Yes: 28 (27.5); No: 2 (1.96); N/A: 72 (70.6)
Does the *P* value demonstrate significance (*P*<.05)?	Yes: 1 (50); No: 0 (0); N/A: 1 (50)	Yes: 35 (10.5); No: 1 (0.301); N/A: 296 (89.2)	Yes: 27 (26.5); No: 1 (0.980); N/A: 74 (72.5)
Does the *P* value demonstrate near significance (*P*<.2)?	Yes: 0 (0); No: 0 (0); N/A: 2 (100)	Yes: 1 (0.301); No: 3 (0.904); N/A: 328 (98.8)	Yes: 1 (0.980); No: 1 (0.980); N/A: 100 (98)
Is there a comparator?	Yes: 1 (50); No: 0 (0); N/A: 1 (50)	Yes: 28 (8.43); No: 18 (5.42); N/A: 286 (86.1)	Yes: 26 (25.5); No: 4 (3.92); N/A: 72 (70.6)
Is the comparator validated?	Yes: 0 (0); No: 1 (50); N/A: 1 (50)	Yes: 9 (2.71); No: 21 (6.33); N/A: 302 (91.0)	Yes: 9 (8.82); No: 17 (16.7); N/A: 76 (74.5)

a Not applicable.

### What Are Mental Health Apps’ Use and Development Characteristics

Table 8 depicts the characteristics of mental health apps regarding their use and development. This table states what key information is available to users of digital mental health apps and whether key professionals were involved. This study shows that 241 out of 436 (55.3%) apps included a qualified professional in app development. A total of 251 out of 436 (57.6%) apps provided evidence within the app that the developer validated any guidance with relevant reliable information sources or references.

**Table 8.** Mental health apps characteristics regarding their use and development.

Question	MH^a^ apps that answered the question, n	Yes, n (%)	No, n (%)
Is there a suitably qualified professional involved in the development team of the app?	436	241 (55.3)	195 (44.7)
Does the organization behind the app have relevant credentials?	436	26 (5.96)	410 (94)
Is there evidence of an endorsement by a relevant body?	436	78 (17.9)	358 (82.1)
Are organizations using the app?	436	110 (25.2)	326 (74.8)
Is there a statement that it has been positively evaluated or validated by a relevant health care professional?	436	76 (17.4)	360 (82.6)
Is there evidence within the app that the developer has validated any guidance with relevant reliable information sources or references?	436	251 (57.6)	185 (42.4)
Is there a statement or any evidence showing that appropriate safeguarding measures are in place around peer-support and other communication functions within the platform?	171	141 (82.5)	30 (17.5)
Does the developer make clear risks associated with using the app?	436	27 (6.19)	409 (93.8)
Is there a privacy policy clearly available via the app/web app/website?	374	364 (97.3)	10 (2.67)
Does the developer fully inform the user of how they will collect data about them?	407	376 (92.4)	31 (7.62)
Is there a statement that confirms the app’s compliance to GDPR^b^ 2016 (or Data Protection Act 2018)?	371	160 (43.1)	211 (56.9)
Are users informed of their rights with regard to their data? Are users clearly informed of the individual privacy rights they are entitled to expect under GDPR?	365	270 (74)	95 (26)

a MH: mental health.

b GDPR: General Data Protection Regulation.

### What Data Is Typically Collected by Mental Health Apps?

**Figure 6.**
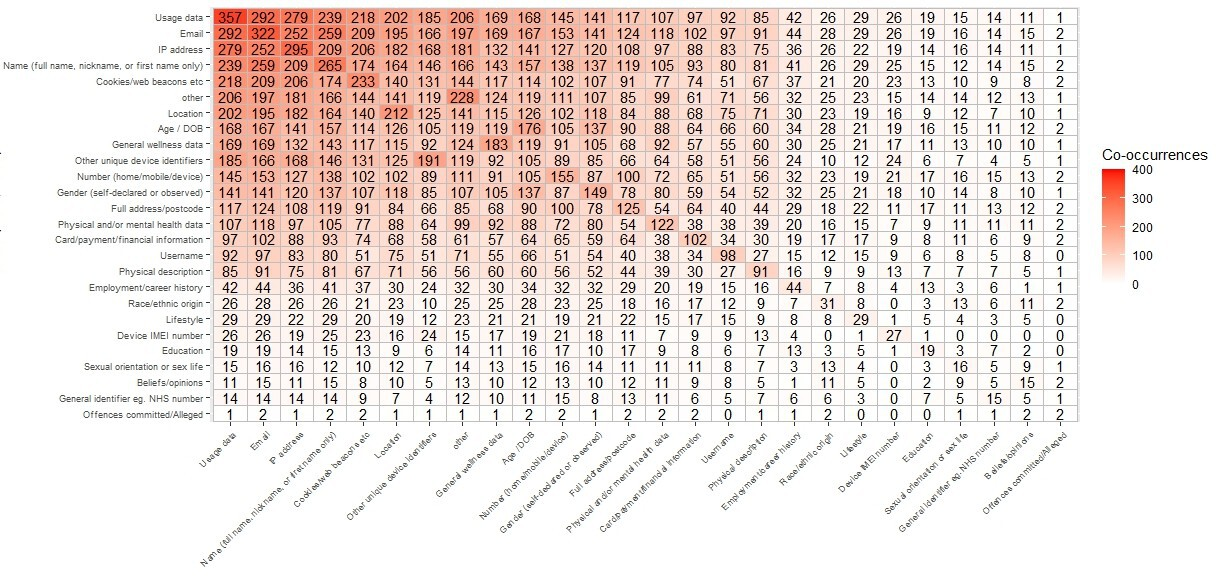
Heatmap of occurrences of collected data. Apps with “No information on data collection provided” and “None (data is stored on the device).” A total of 399 mental health apps are represented in the figure. DOB: date of birth; IMEI: International Mobile Equipment Identity.

Table 9 depicts the types of data that mental health apps collect. Non–mental health apps have been included as a comparator. The table is sorted in descending order of digital mental health app frequency. The most common data type is usage data (357/436, 81.9%), followed by email (322/436, 73.9%). Percentage delta indicates the difference between mental health apps and non–mental health apps. The Fisher exact test (with Bonferroni-corrected α) was used to check for statistical significance between mental health apps and non–mental health apps. Many mental health apps collected data that may not be necessary to provide a mental health service. These were cookies or web beacons (233/436, 53.4%), location data (212/436, 48.6%), and full address or postcode (125/436, 28.7%). However, non–mental health apps also collected these data with a similar proportion; see Table 9 for a list of what types of data were collected. Figure 6 depicts occurrences of data types that were collected by the same apps. Multimedia Appendix 4 depicts the top 10 rules resulting from an association rule mining analysis, showing that email, IP address, name, and usage data are often co-collected by the same apps.

Figure 6 depicts occurrences of data types that were collected together by digital mental health apps. The most common occurrences appear in the top left.

**Table 9.** Mental health apps data collection. Bonferroni corrected α=0.002.

Data type	Frequency of MH^a^ apps (N=436), n (%)	Frequency of all other apps (N=1148), n (%)	Delta (difference), %	*P* value (Fisher exact test)
Usage data	357 (81.9)	860 (74.9)	7	.003
Email	322 (73.9)	792 (69)	4.9	.07
IP address	295 (67.7)	680 (59.2)	8.5	.002
Name (full name, nickname, or first name only)	265 (60.8)	727 (63.3)	2.5	.35
Cookies/web beacons, etc (used for tracking an individual’s online browsing behaviors/movements)	233 (53.4)	576 (50.2)	3.2	.26
Other	228 (52.3)	573 (49.9)	2.4	.40
Location	212 (48.6)	487 (42.4)	6.2	.03
Other unique device identifiers	191 (43.8)	432 (37.6)	6.2	.03
General wellness data	183 (42)	419 (36.5)	5.5	.049
Age/DOB^b^	176 (40.4)	515 (44.9)	4.5	.11
Number (mobile number/device number/home phone number)	155 (35.6)	446 (38.9)	3.3	.25
Gender (self-declared or observed)	149 (34.2)	430 (37.5)	3.3	.24
Full address/postcode	125 (28.7)	355 (30.9)	2.2	.39
Physical and/or mental health data	122 (28)	413 (36)	8	.003
Card/payment/financial information	102 (23.4)	229 (19.9)	3.5	.15
Username	98 (22.5)	216 (18.8)	3.7	.11
Physical description	91 (20.9)	279 (24.3)	3.4	.16
Employment/career history	44 (10.1)	105 (9.15)	0.95	.56
Race/ethnic origin	31 (7.11)	68 (5.92)	1.19	.42
Lifestyle (marital status/family/social circumstance)	29 (6.65)	75 (6.53)	0.12	.91
No information on data collection provided	28 (6.42)	102 (8.89)	2.47	.12
Device IMEI^c^ number	27 (6.19)	38 (3.31)	2.88	.02
Education (qualifications/professional training/awards)	19 (4.36)	57 (4.97)	0.61	.69
Sexual orientation or sex life	16 (3.67)	46 (4.01)	0.34	.89
General identifier eg, NHS^d^ No	15 (3.44)	54 (4.70)	1.26	.34
Beliefs/opinions (political, religious, or philosophical)	15 (3.44)	15 (1.31)	2.13	.01
None (data are stored on the device)	9 (2.06)	24 (2.09)	0.03	>.99
Offenses committed/alleged (criminal proceedings/outcomes/sentence)	2 (0.459)	1 (0.087)	0.372	.19

a MH: mental health.

b DOB: date of birth.

c IMEI: International Mobile Equipment Identity.

d NHS: National Health Service.

**Figure 7.**
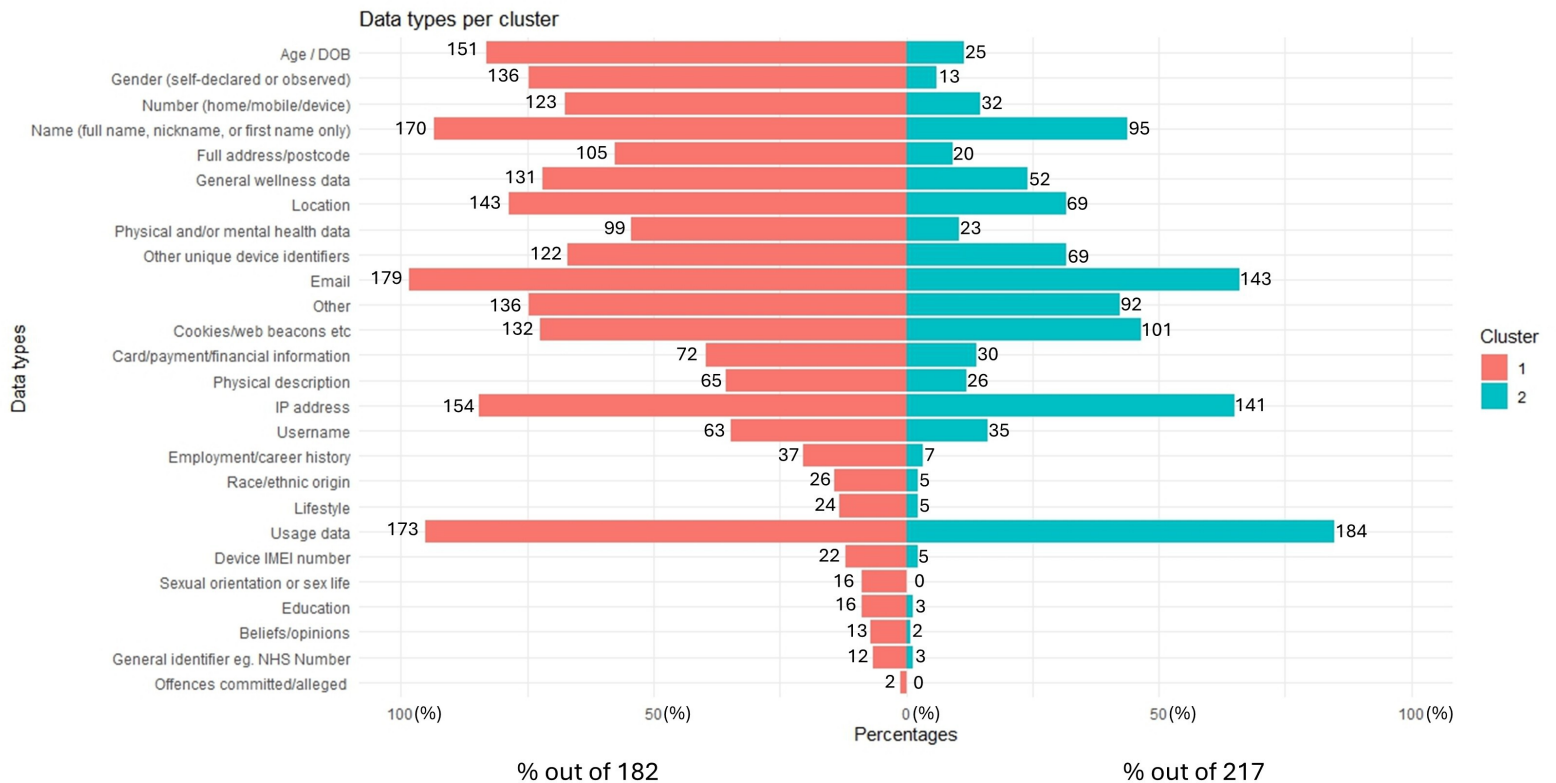
Bar plots showing frequency of data types per cluster, ordered in descending order of percentage delta (% difference) between clusters. DOB: date of birth; IMEI: International Mobile Equipment Identity; NHS: National Health Service.

The results show that mental health apps can be split into 2 clusters. This is visualized in Figure 7, where mental health apps in cluster 1 collected more data than cluster 2. Moreover, on average (median), mental health apps in cluster 1 present a higher ORCHA score than apps in cluster 2. This indicates that mental health apps that collected more data, on average (median), outperformed those that collected less. Previous work analyzed the top 27 ranked Google Play Store mental health apps regarding their DP [26]. It found that many of the apps had DP-related issues such as unnecessary permissions, insecure cryptography implementations, leaks of personal data, credentials in logs, web requests, as well as risk of user profiling. Further analysis is needed to confirm whether it is data collection that contributed to the apps achieving higher ORCHA scores in this study, as indicated by cluster analysis.

K-modes cluster analysis was conducted on 399 of 436 mental health apps (apps with data types “No information on data collection provided” and “None” were excluded). Figure 7 depicts bar plots for each of the data types per cluster. The x-axis represents percentages (corresponding to the size of each cluster) of frequency for each of the data types per cluster. Multimedia Appendix 5 depicts the results of K-modes clustering conducted on the 399 mental health apps.

MultimediaAppendices 4  5 depict the results of association rule mining and K-modes clustering conducted on data types, respectively.

## Discussion

### Principal Findings

The objective of this study was to examine the characteristics of mental health apps. A total of 436 digital mental health apps were assessed in this study. This study found that most mental health apps do not meet the necessary threshold for quality of ORCHA score of 65. Many apps do not follow precautions such as involvement of professionals in the app development process, and organizations behind the apps often do not have the necessary credentials. This study analyzed the distribution of OBR scores across domains (PCA, UX, and DP) and across NICE ESF tiers. The study also analyzed the relationship between quality and cost and the importance of app features in relation to their quality, as indicated by the ORCHA score. Moreover, this study states the types of evidence that underpin mental health apps and the characteristics of app development, use, and DP.

The findings of this study demonstrate that many publicly available mental health apps may not take precautions (such as involvement of appropriate health professionals, literature references, or conducting tests) to ensure that their content is valid and research based. Moreover, as shown by previous research [6-8,18,19], there is a place for improvement regarding the quality of mental health apps. The inclusion of an appropriate health professional in the development of a mental health app could potentially improve the quality of an app, as they would be able to advise on the content of an app and any necessary features.

A study analyzing the mental health app marketplace found that, for mental health apps, user ratings and downloads are not linked with app features, function, or evidence [27]. Similarly, a study that analyzed digital health apps across 26 categories found that user ratings and downloads are not linked to quality [28]. Since user ratings and downloads are not reliable indicators, this study of mental health app characteristics and quality is necessary to further their improvement. Moreover, this indicates that users may be unable to judge whether a mental health app is of appropriate quality and may require expert recommendations. Previous work has been done trying to help in the selection of mental health apps by proposing a digital health app database [29] of apps that were assessed using the American Psychiatric Association app evaluation model [30]. Similarly, the ORCHA database could be used [31], allowing for better selection of digital health apps, which may improve their adoption. A study focused on opportunities and tensions surrounding the use of technology-enabled mental health services found that clinicians saw potential in using technology-enabled mental health care services [32]. In the context of mental health apps, this study could be helpful in making technology-enabled mental health care services improve in quality.

A study on implementation strategies for mental health apps in health care settings [33] explored strategies such as “Exploration Phase Strategies.” This strategy considers the use of evidence-based services based on the needs of those in a setting. This study on the characteristics of mental health apps may influence future strategies, as it provides insight into the quality of mental health apps, the types of evidence that are collected, and the data types that are collected. This study may be useful in determining what is needed for a mental health app to be considered useful and reliable.

### Limitations

The OBR evolved from earlier versions during the height of the COVID-19 pandemic. Originally, it was created as a more stringent version so that ORCHA could recommend the most compliant digital health apps to members of the UK population with confidence. ORCHA tested a new version of the OBR on a selection of highly compliant digital health apps (as determined by prior versions of the OBR). This set of 30 digital health apps served as the pilot group, with the subsequent 2097 (original dataset used in this study, Figure 1) digital health apps being assessed with ORCHA’s typical assessment approach of categorizing digital health apps into categories ordered by number of downloads and assessing the most downloaded digital health app in each category, followed by the second, and so forth.

This study is limited to the characteristics that were included in the OBR assessment, meaning that features not included in the OBR may have a strong influence on quality but were not considered in this study. For example, target users such as gender or mental health symptoms were not included. Future studies could focus on additional characteristics. This study was exploratory; a more in-depth study in terms of target users and features could be conducted, for example, by analyzing feature importance for different categories of mental health apps.

This study was conducted using 436 mental health apps; a study with a larger sample size could lead to better results. Using different assessment tools (other than the OBR) could provide different results, as different characteristics and questions may be included, and a study comparing these findings with findings from other tools could lead to more insights.

### Conclusion

The objective of this study was to examine the characteristics of mental health apps regarding their quality, target users, features, underpinning evidence, and DP. To achieve this objective, 7 exploratory research questions were proposed and answered. In this study, common features and data types were examined regarding their link to quality, as indicated by the ORCHA score. The key findings of the study indicate that the quality of mental health apps could be improved, as the median ORCHA score for mental health apps is 60, which is below the ORCHA threshold score of 65. The findings of this study demonstrate that many publicly available mental health apps did not take precautions (such as involving appropriate health professionals, literature references, or conducting tests) to ensure that their content is valid and research based. Greater effort on behalf of mental health app developers is needed to ensure that the public is provided with high-quality apps.

## Supplementary material

10.2196/67944 Multimedia Appendix 1 Mental health apps’ categories and frequency.

10.2196/67944 Multimedia Appendix 2 Features’ frequency separated by tier.

10.2196/67944 Multimedia Appendix 3 Box plots of the three most important features for each tier against Organisation for the Review of Care and Health Apps (ORCHA) scores.

10.2196/67944 Multimedia Appendix 4 Association rule mining analysis for mental health apps’ data types.

10.2196/67944 Multimedia Appendix 5 K-modes cluster analysis for mental health apps’ data types.

10.2196/67944 Checklist 1 PRISMA checklist.

## Abbreviations

DP: data privacy
ESF: Evidence Standard Framework
NICE: National Institute for Health and Care Excellence
OBR: ORCHA Baseline Review
ORCHA: Organisation for the Review of Care and Health Apps
PCA: professional and clinical assurance
UX: user experience
