# Supplementary material for: The Quality and Characteristics of Digital Mental Health Apps: Mixed Methods Study
Source: JMIR Hum Factors. 2026 May 11;13:e67944. doi: 10.2196/67944 (PMC13160478; doi:10.2196/67944)
Supplement: Multimedia Appendix 1 [file humanfactors-v13-e67944-s001.docx]

**Table S1**: Mental health apps' categories and frequency.

| **Category** | **No. of Apps** |
| --- | --- |
| Anxiety | 229 |
| Stress | 216 |
| Mental Wellbeing | 205 |
| Depression | 105 |
| Relaxation Techniques | 77 |
| Sleep Hygiene | 54 |
| Wellness | 39 |
| Insomnia | 30 |
| Addiction | 26 |
| Eating Disorders | 16 |
| Nutrition | 15 |
| Weight Loss | 15 |
| Fitness | 14 |
| Panic Attack | 13 |
| ADHD | 11 |
| Bipolar Disorder | 11 |
| Obsessive-Compulsive Disorder | 11 |
| PTSD | 11 |
| Self Harm Prevention | 10 |
| Alcohol Awareness | 9 |
| Autistic Spectrum Disorders | 8 |
| COVID-19 | 8 |
| Grief & Bereavement | 7 |
| Suicide Prevention | 7 |
| Dementia | 6 |
| Phobias | 6 |
| Schizophrenia | 6 |
| Type 1 Diabetes | 6 |
| Chronic Pain | 5 |
| Complimentary and Alternative Therapies | 5 |
| Type 2 Diabetes | 5 |
| Children's Health | 4 |
| Caring for Elderly | 3 |
| Early Childhood | 3 |
| Fertility | 3 |
| LGBTIQ+ Support | 3 |
| Pain (generic) | 3 |
| Pregnancy | 3 |
| Psychosis | 3 |
| Smoking Cessation | 3 |
| Social Support Network | 3 |
| Abnormal Heart Rhythms (Arrhythmias) | 2 |
| Arthritis | 2 |
| Atrial Fibrillation | 2 |
| Back Pain | 2 |
| Borderline Personality Disorder | 2 |
| COPD | 2 |
| Child Development | 2 |
| Contraception (Safer Sex) | 2 |
| Diet and Lifestyle During Pregnancy | 2 |
| Dry Eyes | 2 |
| Food Allergy | 2 |
| Heart Failure | 2 |
| High Blood Pressure (Hypertension) | 2 |
| Loneliness | 2 |
| Low Blood Pressure (Hypotension) | 2 |
| Menopause | 2 |
| Polycystic Ovary Syndrome (PCOS) | 2 |
| Social Prescribing | 2 |
| Acne | 1 |
| Alzheimer's | 1 |
| Asthma | 1 |
| Breast feeding | 1 |
| Cancer | 1 |
| Carer resources | 1 |
| Clinical Reference | 1 |
| Communication and Memories | 1 |
| Crohn's Disease | 1 |
| Endometriosis | 1 |
| Epilepsy | 1 |
| Fibromyalgia | 1 |
| Find a Doctor | 1 |
| Incontinence | 1 |
| Irritable Bowel Syndrome | 1 |
| LPP | 1 |
| Memory Training | 1 |
| Multiple Sclerosis | 1 |
| Myalgic Encephalomyelitis/Chronic Fatigue Syndrome | 1 |
| Neurological Disorders | 1 |
| Osteoarthritis | 1 |
| Palliative Care | 1 |
| Paternity | 1 |
| Period Monitoring | 1 |
| Personal Health Record | 1 |
| Psoriasis | 1 |
| Rheumatoid Arthritis | 1 |
| Sexual health | 1 |
| Sexually Transmitted Infection (STI) | 1 |

| **Category** | **Frequency** |
| --- | --- |
| Healthy Living | 381 |
| Medicines and Clinical Reference | 144 |
| Neurological | 128 |
| Pregnancy | 78 |
| Diabetes | 75 |
| Respiratory | 65 |
| Child Health | 64 |
| Women's Health | 63 |
| Utilities/ Administration | 54 |
| Ophthalmology | 54 |
| Sexual Health | 53 |
| Cancer | 52 |
| Musculoskeletal | 49 |
| Neurodiverse | 37 |
| Pain Management | 35 |
| Cardiology | 30 |
| Dermatology | 27 |
| Dental | 25 |
| Ear/Nose/Throat/Mouth | 23 |
| Gastrointestinal | 21 |
| Urology | 14 |
| First Aid | 14 |
| Social support network | 12 |
| Allergy | 12 |
| Older people | 11 |
| Workforce monitoring | 6 |
| Social Prescribing | 5 |
| LGBTIQ+ | 3 |
| Hormone | 3 |
| Blood | 3 |
| Tropical diseases | 2 |
| Men's Health | 2 |

**Table S2**: Categories of digital health apps (Excluding mental health apps).
