# Supplementary material for: The Quality and Characteristics of Digital Mental Health Apps: Mixed Methods Study
Source: JMIR Hum Factors. 2026 May 11;13:e67944. doi: 10.2196/67944 (PMC13160478; doi:10.2196/67944)
Supplement: Multimedia Appendix 2 [file humanfactors-v13-e67944-s002.docx]

**Table S1**: Features for Tier A (2 apps).

| **Features** | **Frequency** |
| --- | --- |
| Information Provision | 1 |
| Data Capture | 1 |
| Data Sharing | 1 |

**Table S2**: Features for Tier B (332 apps).

| **Features** | **Frequency** |
| --- | --- |
| Information Provision | 330 |
| Data Capture | 328 |
| Data Sharing | 318 |
| Health Monitoring | 182 |
| Goal Setting and Gamification | 127 |
| Service Signposting | 105 |
| Condition Management | 42 |
| Remote Monitoring | 41 |
| Online Consultation | 38 |
| Behavioural Change Techniques | 24 |
| Remote Clinical Monitoring | 5 |
| Utility / Administrative | 2 |
| Risk Indicator | 1 |
| Online Prescriptions | 1 |
| Environmental Data | 1 |


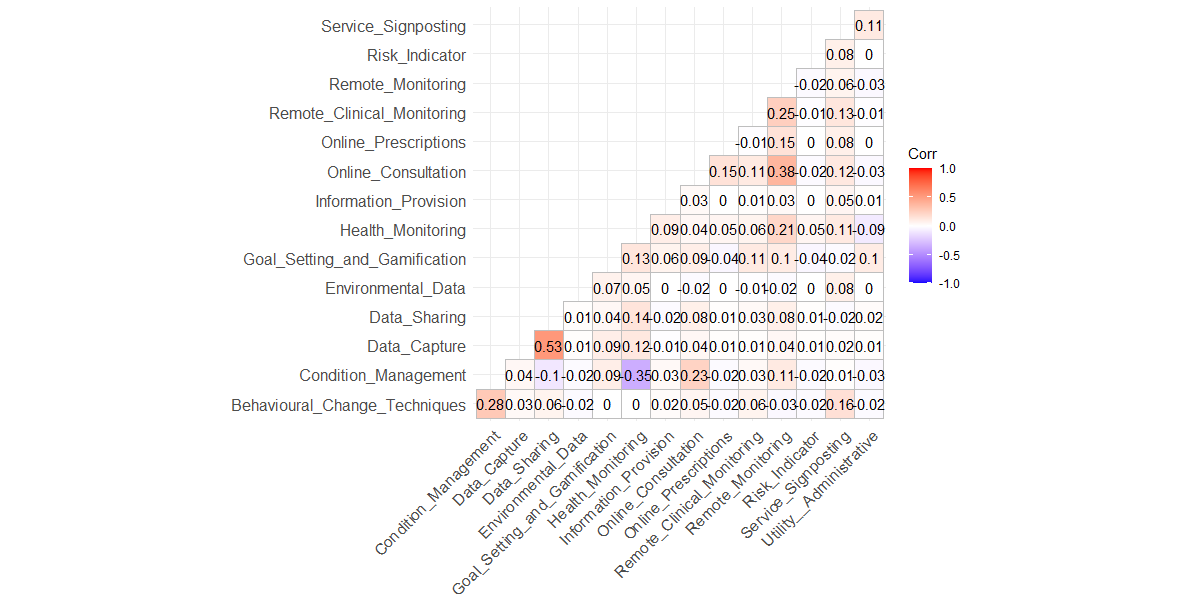


**Figure S1**: Tier B spearman correlation among the features.


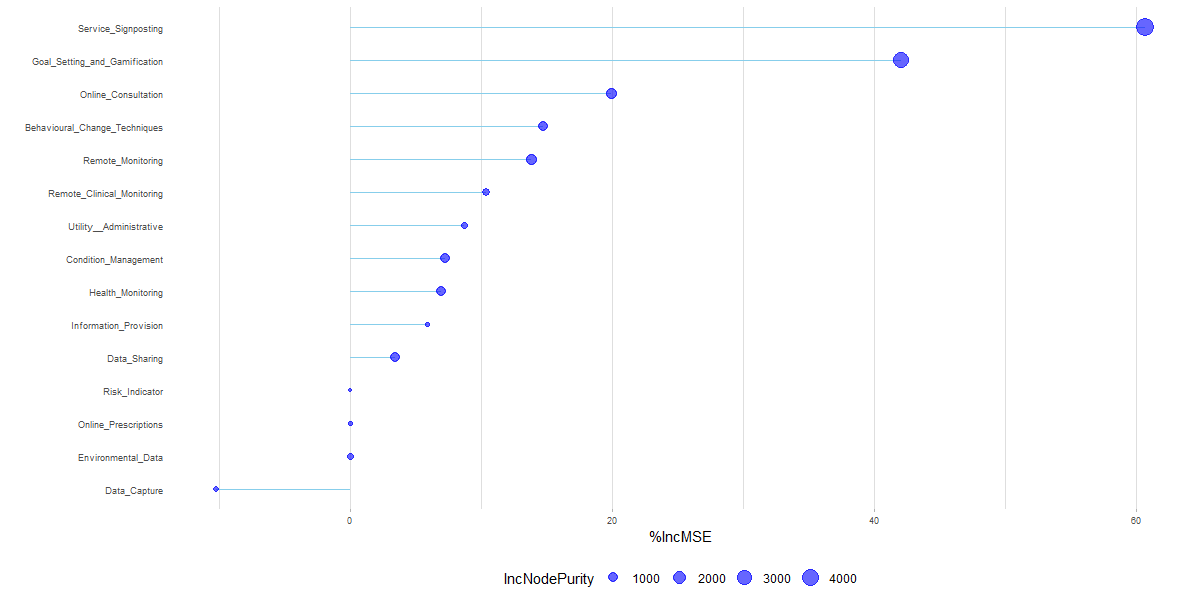


**Figure S 2**: Tier B feature importance with random forest for ORCHA score.

**Table S3**: Features for Tier C (102 apps).

| **Features** | **Frequency** |
| --- | --- |
| Information Provision | 102 |
| Data Capture | 102 |
| Data Sharing | 102 |
| Condition Management | 65 |
| Goal Setting and Gamification | 57 |
| Service Signposting | 54 |
| Health Monitoring | 33 |
| Risk Indicator | 33 |
| Online Consultation | 31 |
| Behavioural Change Techniques | 32 |
| Remote Monitoring | 21 |
| Remote Clinical Monitoring | 12 |
| Treatment Support | 6 |
| Diagnostic Support | 6 |
| Treatment Delivery | 1 |


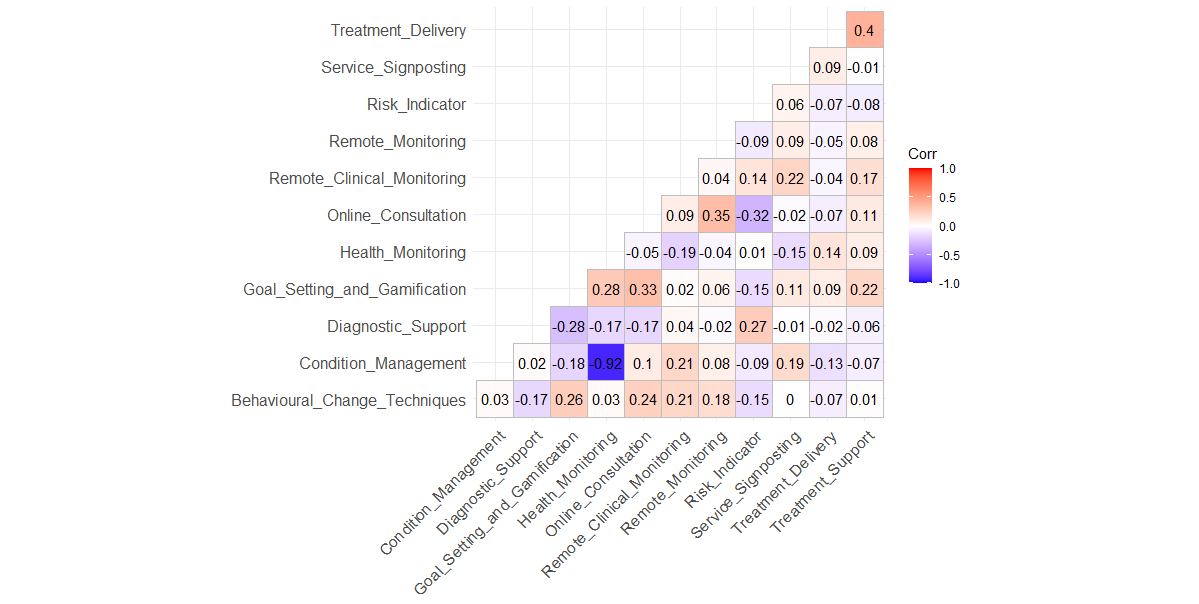


**Figure S3**: Tier C spearman correlation among the features.


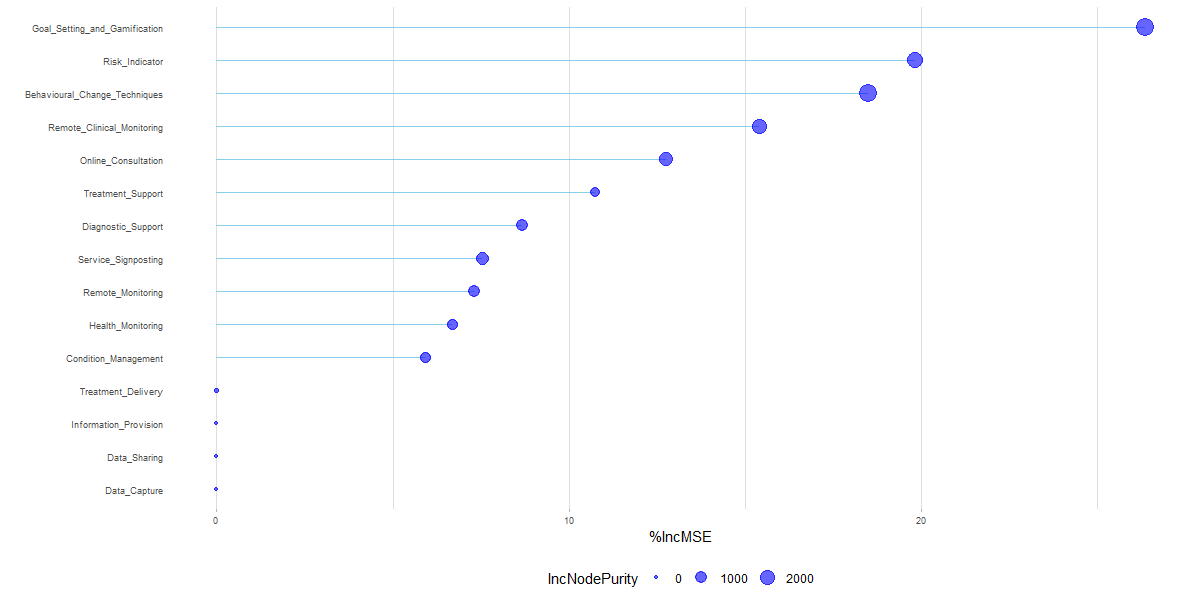


**Figure S4**: Tier C feature importance with random forest for ORCHA score.
