# Supplementary material for: The Quality and Characteristics of Digital Mental Health Apps: Mixed Methods Study
Source: JMIR Hum Factors. 2026 May 11;13:e67944. doi: 10.2196/67944 (PMC13160478; doi:10.2196/67944)
Supplement: Multimedia Appendix 3 [file humanfactors-v13-e67944-s003.docx]

#
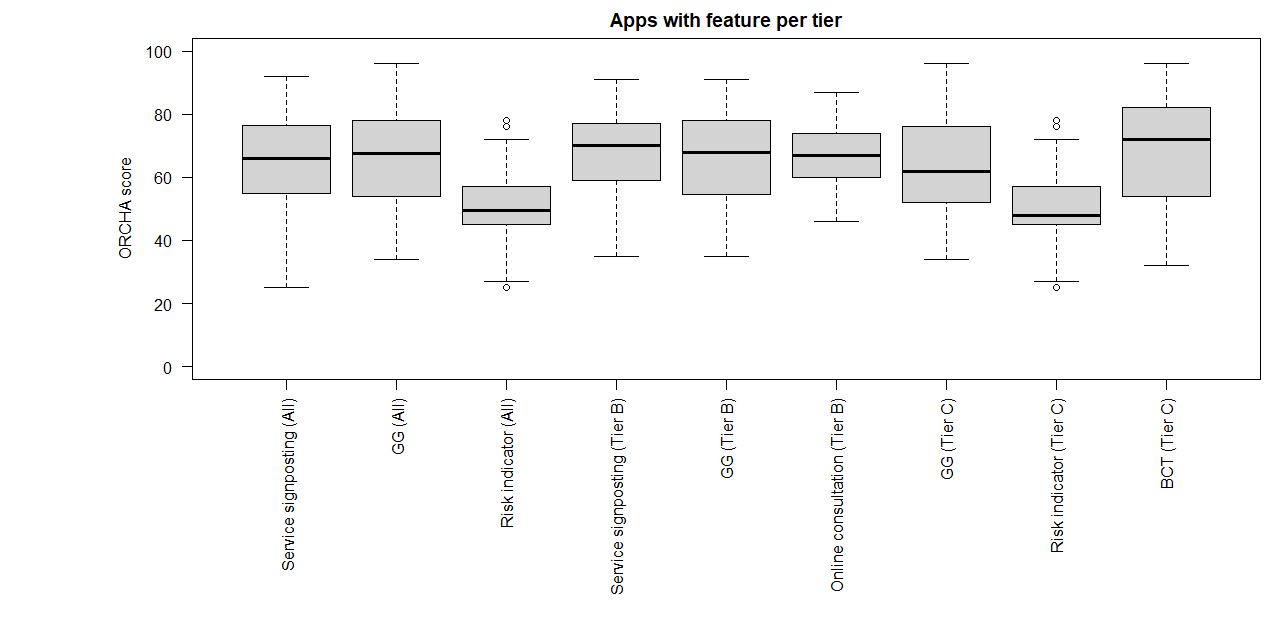


**Figure S1**: ORCHA score of apps with feature per tier.


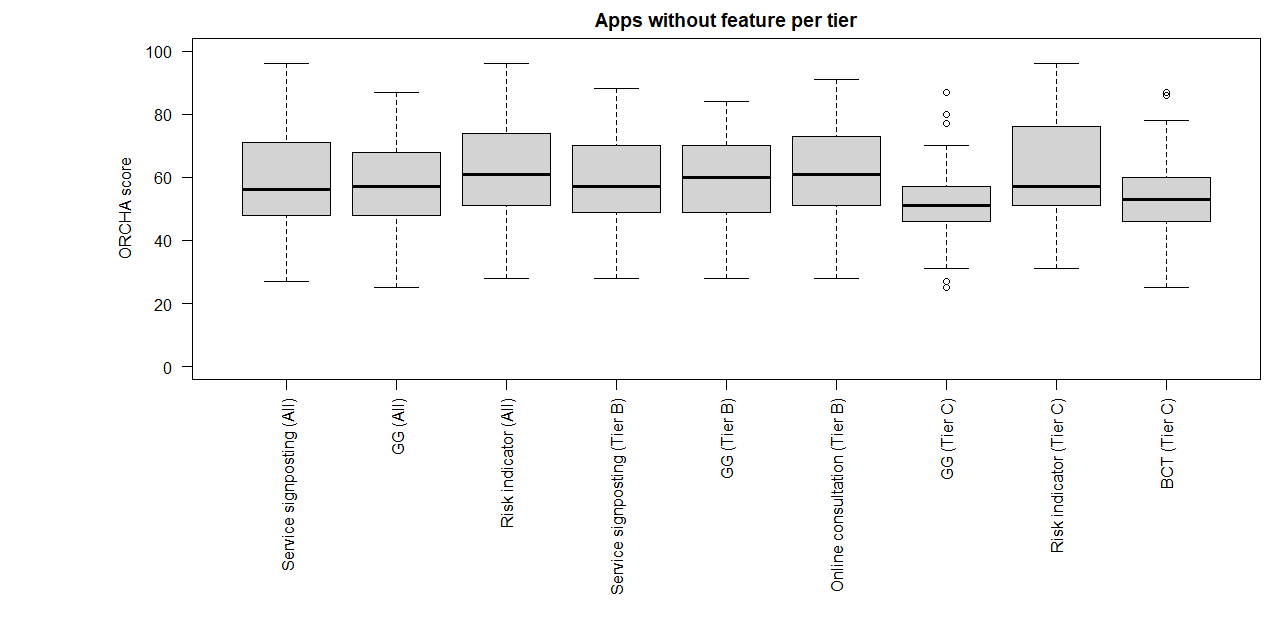


**Figure S2**: ORCHA score of apps without feature per tier.
