# Supplementary material for: The Quality and Characteristics of Digital Mental Health Apps: Mixed Methods Study
Source: JMIR Hum Factors. 2026 May 11;13:e67944. doi: 10.2196/67944 (PMC13160478; doi:10.2196/67944)
Supplement: Multimedia Appendix 4 [file humanfactors-v13-e67944-s004.docx]

**Table S1**: Top 10 association rules with Apriori algorythm among collected data. Ordered in the descending order of lift.

|  | **LHS** | **RHS** | **Support** | **Confidence** | **Coverage** | **Lift** | **Count** |
| --- | --- | --- | --- | --- | --- | --- | --- |
| 1 | {Email, IP Address} | {Name} | 0.5187970 | 0.8214286 | 0.6315789 | 1.236792 | 207 |
| 2 | {IP Address, Name} | {Email} | 0.5187970 | 0.9904306 | 0.5238095 | 1.227273 | 207 |
| 3 | {Usage Data, Name} | {Email} | 0.5864662 | 0.9790795 | 0.5989975 | 1.213207 | 234 |
| 4 | {Email} | {Name} | 0.6491228 | 0.8043478 | 0.8070175 | 1.211075 | 259 |
| 5 | {Name} | {Email} | 0.6491228 | 0.9773585 | 0.6641604 | 1.211075 | 259 |
| 6 | {Usage Data, Email} | {Name} | 0.5864662 | 0.8013699 | 0.7318296 | 1.206591 | 234 |
| 7 | {Cookies / Web Beacons etc} | {IP Address} | 0.5162907 | 0.8841202 | 0.5839599 | 1.195810 | 206 |
| 8 | {IP Address} | {Cookies / Web Beacons etc} | 0.5162907 | 0.6983051 | 0.7393484 | 1.195810 | 206 |
| 9 | {Usage Data, Email} | {IP Address} | 0.6015038 | 0.8219178 | 0.7318296 | 1.111679 | 240 |
| 10 | {Cookies / Web Beacons etc} | {Email} | 0.5238095 | 0.8969957 | 0.5839599 | 1.111495 | 209 |
