# Supplementary material for: The Quality and Characteristics of Digital Mental Health Apps: Mixed Methods Study
Source: JMIR Hum Factors. 2026 May 11;13:e67944. doi: 10.2196/67944 (PMC13160478; doi:10.2196/67944)
Supplement: Multimedia Appendix 5 [file humanfactors-v13-e67944-s005.docx]

**Table S1** shows results of K-modes clustering on data types collected by mental health apps. Number of clusters was chosen using NbClust package in R, out of 30 indices, 10 indices propossed 2 clusters followed by 8 indices that proposed 4 clusters. Based on these results 2 clusters had been chosen to conduct the analysis.

**Table S1**: K-modes clusters. Bonferroni corrected alpha value .002.

| **Data type** | **Cluster 1** | **Cluster 2** | ***Delta %*** | ***p*-value (Fisher)** | **Effect size (Cohen W)** |
| --- | --- | --- | --- | --- | --- |
| Usage Data | 173 (95.1%) | 184 (84.8%) | 10.3% | <.001 | .167 |
| Email | 179 (98.4%) | 143 (65.9%) | 32.5% | <.001 | .410 |
| IP Address | 154 (84.6%) | 141 (65.0%) | 19.6% | <.001 | .223 |
| Name (Full Name, Nickname or First Name Only) | 170 (93.4%) | 95 (43.8%) | 49.6% | <.001 | .523 |
| Cookies / Web Beacons etc. (used for tracking an individual’s online browsing behaviours/movements) | 132 (72.5%) | 101 (46.5%) | 26.0% | <.001 | .263 |
| Other | 136 (74.7%) | 92 (42.4%) | 32.3% | <.001 | .325 |
| Location | 143 (78.6%) | 69 (31.8%) | 46.8% | <.001 | .467 |
| Other Unique Device Identifiers | 122 (67.0%) | 69 (31.8%) | 35.2% | <.001 | .351 |
| General Wellness Data | 131 (72.0%) | 52 (24.0%) | 48.0% | <.001 | .480 |
| Age / DOB | 151 (83.0%) | 25 (11.5%) | 71.5% | <.001 | .717 |
| Number (Mobile Number / Device Number / Home Phone Number) | 123 (67.6%) | 32 (14.7%) | 52.9% | <.001 | .540 |
| Gender (self-declared or observed) | 136 (74.7%) | 13 (5.99%) | 68.7% | <.001 | .708 |
| Full Address/Postcode | 105 (57.7%) | 20 (9.22%) | 48.5% | <.001 | .521 |
| Physical and/or Mental Health Data | 99 (54.4%) | 23 (10.6%) | 43.8% | <.001 | .474 |
| Card/Payment/Financial Information | 72 (39.6%) | 30 (13.8%) | 25.8% | <.001 | .294 |
| Username | 63 (34.6%) | 35 (16.1%) | 18.5% | <.001 | .214 |
| Physical Description | 65 (35.7%) | 26 (12.0%) | 23.7% | <.001 | .282 |
| Employment / Career History | 37 (20.3%) | 7 (3.23%) | 17.1% | <.001 | .272 |
| Race / Ethnic Origin | 26 (14.3%) | 5 (2.30%) | 12.0% | <.001 | .223 |
| Lifestyle (Marital Status / Family / Social Circumstance) | 24 (13.2%) | 5 (2.30%) | 10.9% | <.001 | .209 |
| Device IMEI Number | 22 (12.1%) | 5 (2.30%) | 9.80% | <.001 | .194 |
| Education (Qualifications / Professional Training / Awards) | 16 (8.79%) | 3 (1.38%) | 7.41% | .001 | .173 |
| Sexual Orientation or Sex Life | 16 (8.79%) | 0 (0%) | 8.79% | <.001 | .223 |
| General Identifier eg. NHS No | 12 (6.59%) | 3 (1.38%) | 5.21% | .008 | .136 |
| Beliefs/Opinions (Political, Religious or Philosophical) | 13 (7.14%) | 2 (0.922%) | 6.22% | .001 | .1629 |
| Offences Committed / Alleged (Criminal Proceedings / Outcomes / Sentence) | 2 (1.10%) | 0 (0%) | 1.10% | .207 | .078 |
| **Median ORCHA score (IQR)** | 67.5(21) | 56(22) |  |  |  |
| **Cluster size (% out of 399)** | 182 (45.6%) | 217  (54.4%) |  |  |  |
